# Synthesis and Structure-Activity Relationships of LP1 Derivatives: *N*-Methyl-*N*-phenylethylamino Analogues as Novel MOR Agonists

**DOI:** 10.3390/molecules23030677

**Published:** 2018-03-16

**Authors:** Rita Turnaturi, Carmela Parenti, Orazio Prezzavento, Agostino Marrazzo, Paschalina Pallaki, Zafiroula Georgoussi, Emanuele Amata, Lorella Pasquinucci

**Affiliations:** 1 Department of Drug Sciences, Medicinal Chemistry Section, University of Catania, Viale A. Doria 6, 95125 Catania, Italy; rita.turnaturi@unict.it (R.T.); prezzave@unict.it (O.P.); marrazzo@unict.it (A.M.); eamata@unict.it (E.A.); 2 Department of Drug Sciences, Pharmacology and Toxicology Section, University of Catania, Viale A. Doria 6, 95125 Catania, Italy; cparenti@unict.it; 3 Laboratory of Cellular Signaling and Molecular Pharmacology, Institute of Biosciences and Applications, National Centre for Scientific Research “Demokritos” Agia Paraskevi-Attikis, 15310 Athens, Greece; ppallaki@bio.demokritos.gr (P.P.); iro@bio.demokritos.gr (Z.G.)

**Keywords:** 6,7-benzomorphan derivatives, MOR agonist, pain, radioligand competitive binding, cAMP accumulation assay, tail-flick test

## Abstract

The opioid pharmacological profile of *cis*-(−)-*N*-normetazocine derivatives is deeply affected by the nature of their *N*-substituents. Here, our efforts were focused on the synthesis and pharmacological evaluation of novel derivatives of the lead LP1, a multitarget opioid analgesic compound featuring an *N*-phenylpropanamido substituent. LP1 derivatives **5a**–**d** and **6a**–**d** were characterized by flexible groups at the *N*-substituent that allow them to reposition themselves relative to *cis*-(−)-*N*-normetazocine nucleus, thus producing different pharmacological profiles at the mu, delta and kappa opioid receptors (MOR, DOR and KOR) in in vitro and in vivo assays. Among the series, compound **5c**, with the best in vitro and in vivo profile, resulted a MOR agonist which displays a K_i_^MOR^ of 6.1 nM in a competitive binding assay, and an IC_50_ value of 11.5 nM and an I_max_ of 72% in measurement of cAMP accumulation in HEK293 cells stably expressing MOR, with a slight lower efficacy than LP1. Moreover, in a mouse model of acute thermal nociception, compound **5c,** intraperitoneally administered, exhibits naloxone-reversed antinociceptive properties with an ED_50_ of 4.33 mg/kg. These results expand our understanding of the importance of *N*-substituent structural variations in the opioid receptor profile of *cis*-(−)-*N*-normetazocine derivatives and identify a new MOR agonist useful for the development of novel opioid analgesics for pain treatment.

## 1. Introduction

The *cis*-(−)-*N*-normetazocine nucleus, derived by structure-activity relationships (SARs) during morphine skeleton simplification [1,2], has been the subject of medicinal chemistry exploration for the development of new drugs and pharmacological tools to explore opioid pharmacology in vitro and in vivo [3]. This nucleus is a rigid scaffold able to support the phenolic ring and the basic nitrogen in a correct conformation mimicking the tyramine of opioid peptides [4]. A plethora of experimental evidence demonstrated the unpredictability of agonist and antagonist properties conferred by *N*-substituents on 6,7-benzomorphan ligands [5,6,7,8,9]. Thus, our approach consisted in the introduction at the basic nitrogen of the *cis*-(−)-*N*-normetazocine nucleus of different functional groups with the aim to explore their influence on the mu, delta and kappa opioid receptor (MOR, DOR and KOR) affinity, selectivity and activity in the resulting compounds. 

**Figure 1:**
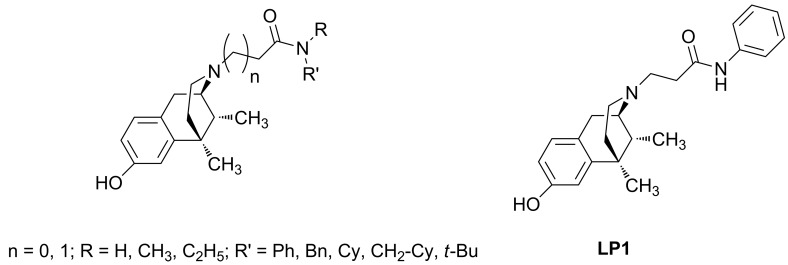
Structures of LP1 and its analogues.

Previously, we synthesized a series of compounds by introducing at the *N*-substituent of *cis*-(−)-*N*-normetazocine an aromatic ring and/or alkyl residues linked by an *N*-propanamido or *N*-acetamido spacers (Figure 1) [10]. Among them, the multitarget opioid ligand LP1, featured by an *N*-phenylpropanamido substituent, possessed affinity and intrinsic activity for MOR (K_i_ = 0.83 nM, IC_50_ = 4.8 nM) and DOR (K_i_ = 29.1 nM, IC_50_ = 12 nM). LP1, in vivo tested, resulted a potent opioid analgesic compound (ED_50_ = 2.03 mg/kg) with low tolerance-inducing capability [11,12] also suitable for the management of persistent pain conditions such as neuropathic and inflammatory pain [13]. 

**Figure 2:**
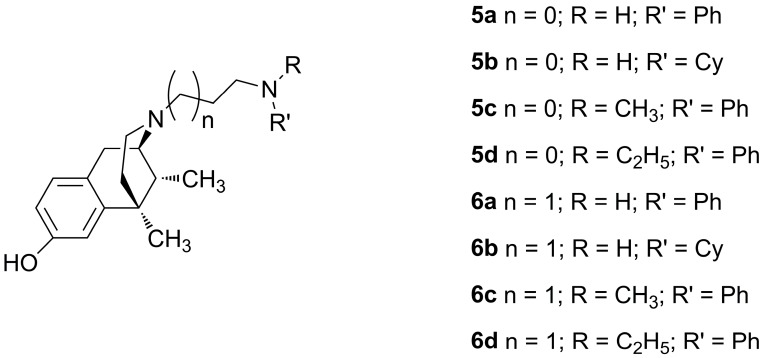
Structures of LP1 *N*-modified derivatives **5a**–**d** and **6a**–**d**.

Here, our efforts were focused on the evaluation of the effect of *N*-substituent structural variations on the opioid receptor profile of LP1 through the design and synthesis of two new series of derivatives. The new LP1 derivatives designed possessed different secondary and tertiary ethylamino or propylamino side chains bearing phenyl or cyclohexyl rings (Figure 2). Thus, they may help our understanding of opioid ligand-receptor interactions. All synthesized LP1 derivatives were tested by opioid receptor competitive binding assays to determine their affinity at MOR, DOR and KOR. Moreover, the compounds were further evaluated in vitro by measuring their adenylyl cyclase (AC) activity in order to establish their functional profile. Finally, the compound with the best functional profile was also tested in vivo through a mouse tail flick test.

In the new synthesized benzomorphan ligands flexible groups at the *N*-substituent position allow them to reposition themselves relative to the *cis*-(−)-*N*-normetazocine nucleus thus producing a different pharmacological profile at MOR, DOR and KOR. In particular, compound **5c**, with the best in vitro and in vivo profile, resulted a MOR agonist suitable for the development of novel analgesic drugs. 

## 2. Results and Discussion

### 2.1. Chemistry

**Scheme 1:**
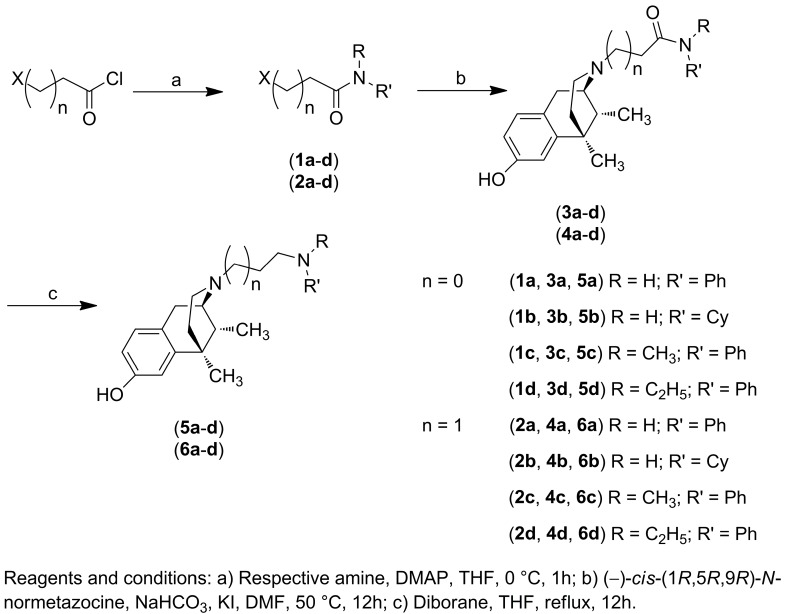
Synthetic pathway.

The synthesis of the novel compounds **5a**–**d** and **6a**–**d** was carried out as illustrated in Scheme 1. Amides **1a**–**d** and **2a**–**d** were prepared by acylation of the respective amines with 2-chloroacetyl chloride and 3-bromopropionyl chloride, respectively, and compounds **3a**–**d** and **4a**–**d** were obtained by alkylation of *cis*-(−)-(1*R*,5*R*,9*R*)-*N*-normetazocine with the respective amides, as previously reported [10,14]. Then, reduction of compounds **3a**–**d** and **4a**–**d** with diborane in anhydrous THF provided the final compounds **5a**–**d** and **6a**–**d**. The new synthesized derivatives were characterized by IR, ^1^H-NMR, ^13^C-NMR and elemental analysis.

### 2.2. Pharmacology

#### 2.1.1. In Vitro Radioligand Binding Assay

The MOR, DOR and KOR binding affinity of compounds **5a**–**d** and **6a**–**d** were determined by radioligand competitive binding experiments as previously reported [15]. Calculated inhibition constant (K_i_) values are listed in Table 1.

As shown in Table 1, LP1 derivatives displayed a broad range of binding affinity for MOR (K_i_ = 6.1–160 nM) and KOR (K_i_ = 19.8–56 nM) and lesser or no affinity towards DOR. The first series of ligands **5a**–**d**, with an *N*-ethylamino spacer, retained a significant MOR affinity, in particular **5c** and **5b**, although in comparison to the lead LP1 their K_i_ values were 7- and 10-times higher. With the exception of compound **5a**, an improved KOR affinity was reported for compounds **5b**–**d**. Indeed, their K_i_ values were 5-, 3- and 4-times lower than the LP1 K_i_ value. A worse DOR affinity profile was recorded for compounds **5a**–**d**. 

For the second series of ligands **6a**–**d**, with an *N*-propylamino side chain, a similar trend of affinity was recorded. The binding profile of compounds **6a**–**d** versus MOR got worse, and in fact their K_i_ values were 10-, 58-, 82- and 103 times higher in comparison to LP1. In addition, in comparison with the series containing the *N*-ethylamino substituent, the K_i_ values of compounds **6a**–**d** for MOR were higher. Analogously to the series **5a**–**d**, an improved KOR binding affinity was recorded also for the series with the *N*-propylamino substituent **6a**–**d**. Their K_i_ values for KOR were lower in comparison to LP1 and higher in comparison to the series **5a**–**5d**. The introduction of the *N*-propylamino substituent was significantly detrimental for DOR binding affinity. 

In general, in *N*-amino derivatives **5a**–**d** and **6a**–**d** the introduction of a second positive charge at the nitrogen substituent retained MOR affinity, improved KOR affinity and dramatically reduced DOR affinity. Among the series, compounds **5b** and **5c**, with the *N*-cyclohexylethylamino and *N*-methyl-*N*-phenylethylamino substituent, respectively, showed a significant MOR (K_i_ = 8.3 and 6.1 nM, respectively) and KOR (K_i_ = 19.8 and 31 nM, respectively) affinity and the compound **6a**, with the *N*-phenylpropanamino substituent, showed similar MOR/KOR binding values of compounds **5b** and **5c** (Table 1). 

In comparison to the respective previously synthesized and in vitro evaluated *N*-amido series (Figure 1), an opposite trend emerged [10]. Indeed, the shorter *N*-acetamido spacer produced a significant loss of MOR affinity relative to their *N*-propanamido homologs. All *N*-acetamido and *N*-propanamido compounds displayed a low KOR affinity with the partial exception of the compound bearing the *N*-ethyl-*N*-phenylpropanamido substituent (K_i_^KOR^ = 70 nM). Moreover, the *N*-secondary amido substituent was preferred for MOR interaction while the *N*-tertiary amido substituent was unfavorable. These evidences suggested that, in comparison to the *N*-acetamido and *N*-propanamido series, the increased flexibility of *N*-substituent of compounds **5a**–**d** and **6a**–**d** and the presence of a second positive charge could allow a different interaction with the opioid receptor binding pocket. In fact, all new synthesized compounds possess orientable groups at the *N*-substituent that allow to reposition themselves relative to the *cis*-(−)-*N*-normetazocine nucleus giving differences in binding versus MOR, DOR and KOR.

#### 2.1.2. Adenylyl Cyclase-Mediated Effects

As typical Gi/o coupled receptor, MOR, DOR and KOR signals are mediated by AC inhibition. Given the significant MOR and moderate KOR affinity, to examine the functional role of target compounds, in opioid receptor signaling, the ability of **5a**–**d** and **6a**–**d** to affect forskolin-stimulated AC activity in HEK293 cells stably expressing the MOR or KOR was tested and the results are reported in Table 2. 

In HEK293 cells stably expressing the MOR, compounds **5a**–**d** and **6a**–**d**, with the exception of compound **6b**, dose-dependently inhibited cAMP accumulation although with marked differences in potency and efficacy. Compound **5c** resulted a MOR agonist that, in comparison to LP1, showed a 2-times higher IC_50_ value but comparable efficacy. Compounds **5a**, **b**, **d** and **6a**, **c**, **d** resulted weak MOR partial agonists with IC_50_ values ranging from 7.4 nM to 74.0 nM and I_max_ values ranging from 28% to 60% inhibition over control. In parallel experiments in HEK293 cells stably expressing KOR, target compounds exhibited a different trend. In particular, **5a**,**b** and **6a**,**b** showed a very low potency, with IC_50_ values ranging from 180 nM to 5000 nM, and efficacy with I_max_ values ranging from 44% to 58% inhibition over control. Compounds **5c**,**d** and **6c**,**d** were not able to dose-dependently inhibit cAMP accumulation and only at very high concentrations determined 30% cAMP inhibition. 

#### 2.1.3. Tail Flick Test

**Figure 3:**
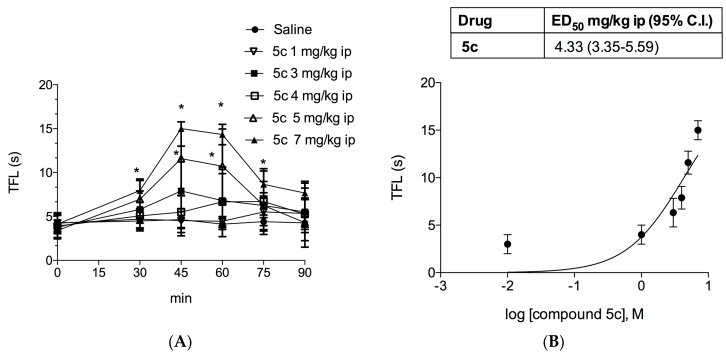
(**A**) Time-course (min) of compound **5c** induced analgesia measured by tail flick latency (TFL). Results are expressed in seconds (s). Data are means ± SEM from six to eight mice. * *p* < 0.05 vs. saline-treated mice. (**B**) Analgesic dose-response curve ± SEM for compound **5c** was plotted at 45 min post-treatment.

The MOR agonist **5c** was evaluated for acute agonistic effects in mice. In detail, it was tested for its ability to produce antinociception in the mouse tail flick test at the dose-range of 1–7 mg/kg intraperitoneal (ip) injected. The compound produced a dose-dependent analgesic effect compared to the group of mice treated with saline (* *p* < 0.05 vs. saline-treated mice). In vivo evaluation of **5c** established that the maximal antinociceptive activity was reached at 45 min (Figure 3, panel A) after ip injection and significantly lasted, at the highest dose, until 75 min after injection. Pretreatment with naloxone (3 mg/kg sc), 30 min prior to **5c**, prevented its antinociceptive effect (data not shown). Figure 3, panel B shows the analgesic dose-response curve with the ED_50_ value (4.33 mg/kg ip) and its confidence limits (CL) (3.35–5.59). These results are in agreement with our in vitro functional assays.

## 3. Discussion

In this study, our attention was focused on the evaluation of *N*-substituents in *cis*-(−)-*N*-normetazocine-based compounds to extend the knowledge about this structural region in the opioid receptor interaction. LP1 derivatives produced a pharmacological profile at MOR, DOR and KOR different one from another as consequence of the *N*-substituent nature whose flexibility allow them to reposition themselves differently relative to the *cis*-(−)-*N*-normetazocine nucleus. Moreover, the presence of a second positive charge at the *N*-substituent, as well as the shortening of the *N*-substituent spacer, seem to address the ligand-opioid receptor interaction mainly at MOR and KOR. Almost all synthesized compounds, in HEK293 cells stably expressing MOR, resulted MOR partial agonists and compound **5c** exhibited an IC_50_ value of 11.5 nM and an I_max_ of 72% with a slight lower efficacy than LP1. In tail flick test compound **5c** confirmed its in vitro profile with an analgesic activity naloxone-reversed and an ED_50_ of 4.33 mg/kg. Moreover, in compound **5c** the second positive charge at the *N*-substituent could affect its pharmacokinetic properties and could, in part, justify its reduced antinociceptive effect compared to LP1 after systemic administration.

Our results corroborated a plethora of experimental data [5,7,8] revealing the unpredictability of pharmacological properties conferred by *N*-substituents on (−)-*cis*-*N*-normetazocine scaffold. For instance, the introduction at the basic nitrogen of an aromatic ring and/or alkyl residues linked by an *N*-acetamido spacer was detrimental for opioid receptors affinity (K_i_^MOR^ 722–2930 nM, K_i_^DOR^ > 5000 nM, K_i_^KOR^ 335–5000 nM) [10]. Contrarily, aromatic ring and/or alkyl residues linked by the *N*-propanamido spacer, as well as the presence of a secondary amido group, improved opioid interaction mainly at MOR [10]. Bulkier aromatic groups, such as naphthyl, quinoline and isoquinoline rings, at the *N*-propanamido spacer, were not tolerated for interaction with the opioid receptors binding pocket mainly at DOR [16]. Moreover, the bulkier size of the *N*-substituent moved the functional profile from agonism to antagonism versus MOR [16]. A shorter and more flexible ethyl spacer with H-bonding groups at carbon 2 in *N*-substituent of (−)-*cis*-*N*-normetazocine nucleus allowed an optimal interaction with opioid binding pocket mainly at MOR and DOR [15].

## 4. Materials and Methods

### 4.1. General Experimental Procedures

All commercial chemicals were purchased from Sigma-Aldrich (St. Louis, MO, USA) or Merck (Darmstadt, Germany) and were used without further purification. (±)-cis-N-normetazocine was obtained from Fabbrica Italiana Sintetici (Milano, Italy). Melting points were determined in open capillary tubes with a Büchi 530 apparatus (Büchi, Flawil, Switzerland) and are uncorrected. Analytical TLC was performed on silica gel 60 F254 aluminum sheets (Merck) with fluorescent indicator. Components were visualized by UV light (λ = 254 nm) and iodine vapors. Flash column chromatography was carried out on Merck silica gel 60 (230–400 mesh). Optical rotations were determined in CHCl_3_ or MeOH solution with a Perkin-Elmer 241 polarimeter (Llantrisant, UK). Infrared spectra were recorded on a 1600 FT-IR Perkin-Elmer instrument. ^1^H- and ^13^C-NMR spectra were routinely recorded on an Inova-200 spectrometer (Varian, Palo Alto, CA, USA) in CDCl_3_ solution; chemical shifts δ are expressed in ppm with reference to tetramethylsilane as an internal standard. Elemental analyses (C, H, N) were performed on a Carlo Erba 1106 analyzer (Milan, Italy) and the analysis results were within ± 0.4% of the theoretical values. All reported compounds had a purity of at least 95%. Synthesis and analytical data of compounds **1a**–**d**, **2a**–**d**, **3a**–**d** and **4a**–**d** were described in reference [10].

### 4.2. Chemistry

*General procedure for the reduction of cis-(−)-N-substituted-N-normetazocine derivatives to afford compounds* **5a***–***d** *and* **6a***–***d**. To a 1 M solution of diborane (2 mL, 2 mmol) in THF, cooled at 0 °C and under nitrogen atmosphere, a solution of the appropriate *cis*-(−)-*N*-substituted-*N*-normetazocine derivatives **3a***–***d** and **4a***–***d** (1 mmol) in THF (4 mL) was slowly added. The reaction mixture was refluxed for 12 h and cooled at room temperature, and 2 mL of a 6 M hydrochloric acid solution was added slowly. THF was removed by distillation at atmospheric pressure. NaHCO_3_ saturated solution was added to aqueous phase, and the latter was extracted with CHCl_3_. The organic mixture was dried over anhydrous Na_2_SO_4_ and evaporated *in vacuo* to give the free base. The compounds **5a***–***d** and **6a***–***d** were dissolved in THF and treated with a solution of H_2_C_2_O_4_⋅2H_2_O in THF to give the oxalate salts as white solids. The analytical pure samples were obtained by recrystallization.

*(2R,6R,11R)-3-(2-Anilinoethyl)-6,11-dimethyl-1,2,3,4,5,6-hexahydro-2,6-methano-3-benzazocin-8-ol* (**5a**). White solid (87%); m.p. 145–146 °C; [α${]}_{D}^{25}$ = −60° (*c* 1.0, MeOH); ^1^H-NMR (CDCl_3_, free base) δ 7.13–7.06 (2H, m), 6.96–6.92 (1H, m), 6.67–6.54 (5H, m), 3.62–3.56 (1H, m), 3.42–3.01 (7H, m), 2.56–2.49 (1H, m), 2.14–1.88 (2H, m), 1.46–1.39 (1H, m), 1.29 (3H, s), 0.79 (3H, d); ^13^C-NMR (CDCl_3_, free base) δ 156.16, 147.90, 140.24, 128.97, 128.37, 123.19, 116.38, 113.74, 112.27, 111.88, 57.95, 51.54, 45.67, 40.15, 39.32, 37.61, 34.70, 28.94, 24.26, 12.93; anal. C 64.72, H 7.21, N 6.35%, calcd. for C_22_H_28_N_2_O⋅C_2_H_2_O_4_⋅H_2_O (444.521), C 64.85, H 7.26, N 6.30%.

*(2R,6R,11R)-3-[2-(Cyclohexylamino)ethyl)-6,11-dimethyl-1,2,3,4,5,6-hexahydro-2,6-methano-3-benzazocin-8-ol* (**5b**). White solid (80%); m.p. 153–155 °C; [α${]}_{D}^{25}$ = −59° (*c* 1.0, MeOH); ^1^H-NMR (CDCl_3_, free base) δ 6.89–6.85 (1H, m), 6.69–6.56 (2H, m), 2.86–2.39 (8H, m), 2.10–1.71 (8H, m), 1.31 (3H, s), 1.28–1.18 (7H, m), 0.80 (3H, d); ^13^C-NMR (CDCl_3_, free base) δ 155.02, 142.95, 127.98, 127.42, 113.27, 112.49, 58.79, 56.98, 53.40, 45.17, 43.20, 42.13, 41.74, 36.36, 32.74, 25.81, 25.45, 24.95, 24.16, 14.08; anal. C 63.94, H 8.52, N 6.27%, calcd for C_22_H_34_N_2_O⋅C_2_H_2_O_4_⋅H_2_O (450.568), C 63.98, H 8.50, N 6.22%.

*(2R,6R,11R)-6,11-Dimethyl-{2-[methyl(phenyl)amino]ethyl}-1,2,3,4,5,6-hexahydro-2,6-methano-3-benzazocin-8-ol* (**5c**). White solid (84%); m.p. 162–163 °C; [α${]}_{D}^{25}$ = −58° (*c* 1.0, MeOH); ^1^H-NMR (CDCl_3_, free base) δ 7.63–7.40 (2H, m), 7.15–6.85 (1H, m), 6.74–6.51 (5H, m), 3.33 (3H, s), 3.26–2.89 (9H, m), 2.57–2.49 (2H, m), 1.33–1.16 (1H, m), 1.29 (3H, s), 0.76 (3H, d); ^13^C-NMR (CDCl_3_, free base) δ 156.29, 148.69, 140.53, 129.52, 128.89, 123.55, 116.93, 114.08, 112.55, 112.14, 59.09, 51.36, 48.95, 46.97, 45.71, 38.24, 35.10, 34.95, 24.47, 23.14, 13.20; anal. C 65.44, H 7.37, N 6.12%, calcd for C_23_H_30_N_2_O⋅C_2_H_2_O_4_⋅H_2_O (458.547), C 65.48, H 7.47, N 6.11%.

*(2R,6R,11R)-6,11-Dimethyl-{2-[ethyl(phenyl)amino]ethyl}-1,2,3,4,5,6-hexahydro-2,6-methano-3-benzazocin-8-ol* (**5d**). White solid (85%); m.p. 158–160 °C; [α${]}_{D}^{25}$ = −59° (*c* 1.0, MeOH); ^1^H-NMR (CDCl_3_, free base) δ 7.15–7.07 (2H, m), 6.86–6.82 (1H, m), 6.63–6.47 (5H, m), 3.50–3.38 (2H, m), 3.31 (2H, q, *J* = 6.8 Hz), 2.84–2.47 (7H, m), 2.17–1.64 (2H, m), 1.42–1.07 (1H, m), 1.25 (3H, s), 1.06 (3H, t, *J* = 7.0 Hz), 0.74 (3H, d); ^13^C-NMR (CDCl_3_, free base) δ 155.51, 147.46, 142.20, 129.11, 127.81, 123.19, 114.93, 112.93, 111.82, 111.28, 60.74, 58.11, 51.81, 45.31, 44.39, 40.21, 39.79, 35.73, 25.23, 23.57, 13.78, 12.17; anal. C 66.17, H 7.66, N 5.95%, calcd. for C_24_H_32_N_2_O·C_2_H_2_O_4_⋅H_2_O (472.574), C 66.08, H 7.68, N 5.93%.

*(2R,6R,11R)-3-(3-Anilinopropyl)-6,11-dimethyl-1,2,3,4,5,6-hexahydro-2,6-methano-3-benzazocin-8-ol* (**6a**). White solid (91%); m.p. 149–150 °C; [α${]}_{D}^{25}$ = −45° (*c* 1.0, MeOH); ^1^H-NMR (CDCl_3_, free base) δ 7.09–7.02 (2H, m), 6.94–6.90 (1H, m), 6.65–6.47 (5H, m), 3.41–3.36 (1H, m), 3.04–2.78 (7H, m), 2.35–2.24 (1H, m), 1.98–1.84 (2H, m), 1.84–1.65 (2H, m), 1.39–1.28 (1H, m), 1.28 (3H, s), 0.77 (3H, d); ^13^C-NMR (CDCl_3_, free base) δ 156.08, 148.75, 140.87, 128.95, 128.75, 128.26, 124.08, 115.69, 113.58, 112.03, 57.55, 51.35, 45.32, 40.57, 39.25, 38.50, 35.16, 24.60, 24.50, 22.85, 13.28; anal. C 65.28, H 7.54, N 6.18%, calcd. for C_23_H_30_N_2_O⋅C_2_H_2_O_4_⋅H_2_O (458.547), C 65.48, H 7.47, N 6.11%.

*(2R,6R,11R)-3-[3-(Cyclohexylamino)propyl)-6,11-dimethyl-1,2,3,4,5,6-hexahydro-2,6-methano-3-benzazocin-8-ol* (**6b**). White solid (94%); m.p. 136–138 °C; [α${]}_{D}^{25}$ = −47° (*c* 1.0, MeOH); ^1^H-NMR (CDCl_3_, free base) δ 6.96–6.92 (1H, m), 6.66–6.59 (2H, m), 3.44–3.41 (1H, m), 2.97–2.88 (6H, m), 2.49–2.35 (1H, m), 2.07–1.98 (7H, m), 1.74–1.56 (3H, m), 1.43–1.28 (6H, m), 1.28 (3H,s), 1.11–1.08 (1H, m), 0.79 (3H, d); ^13^C-NMR (CDCl_3_, free base) δ 156.26, 140.39, 128.35, 123.30, 113.80, 112.00, 60.08, 57.70, 56.04, 55.75, 54.92, 50.13, 45.26, 41.20, 34.87, 28.51, 24.76, 24.36, 23.93, 21.02, 13.09; anal. C 64.55, H 8.64, N 6.09%, calcd. for C_23_H_36_N_2_O⋅C_2_H_2_O_4_⋅H_2_O (464.595), C 64.63, H 8.68, N 6.03%.

*(2R,6R,11R)-6,11-Dimethyl-{3-[methyl(phenyl)amino]propyl}-1,2,3,4,5,6-hexahydro-2,6-methano-3-benzazocin-8-ol* (**6c**). White solid (91%); m.p. 140–142 °C; [α${]}_{D}^{25}$ = −52° (*c* 1.0, MeOH); ^1^H-NMR (CDCl_3_, free base) δ 7.19–7.12 (3H, m), 6.73–6.60 (5H, m), 3.63–3.57 (1H, m), 3.44–2.89 (7H, m), 2.85 (3H, s), 2.49–2.40 (1H, m), 2.09–1.88 (4H, m), 1.45–1.38 (1H, m), 1.28 (3H, s), 0.78 (3H, d); ^13^C-NMR (CDCl_3_, free base) δ 156.29, 148.88, 140.13, 129.04, 128.42, 122.99, 115.96, 113.86, 112.22, 111.99, 57.69, 49.12, 40.75, 39.91, 39.13, 39.00, 38.25, 37.73, 34.77, 24.24, 21.14, 12.92; anal. C 66.10, H 7.64, N 5.99%, calcd. for C_24_H_32_N_2_O⋅C_2_H_2_O_4_⋅H_2_O (472.574), C 66.08, H 7.68, N 5.93%.

*(2R,6R,11R)-6,11-Dimethyl-{3-[ethyl(phenyl)amino]propyl}-1,2,3,4,5,6-hexahydro-2,6-methano-3-benzazocin-8-ol* (**6d**). White solid (93%); m.p. 138–139 °C; [α${]}_{D}^{25}$ = −55° (*c* 1.0, MeOH); ^1^H-NMR (CDCl_3_, free base) δ 7.18–6.92 (3H, m), 6.69–6.52 (5H, m), 3.52–3.37 (2H, m), 3.31 (2H, q, *J* = 6.8 Hz), 2.97–2.91 (4H, m), 2.64–2.45 (3H, m), 2.07–1.74 (4H, m), 1.43–1.36 (1H, m), 1.28 (3H, s), 1.06 (3H, t, *J* = 6.8 Hz), 0.79 (3H, d); ^13^C-NMR (CDCl_3_, free base) δ 156.16, 147.44, 140.44, 129.14, 128.32, 123.48, 115.34, 113.70, 111.95, 111.84, 57.73, 50.53, 46.80, 45.33, 44.07, 39.75, 37.89, 34.93, 25.61, 24.37, 22.56, 13.03, 11.97; anal. C 66.65, H 7.81, N 5.72%, calcd. for C_25_H_34_N_2_O⋅C_2_H_2_O_4_⋅H_2_O (486.601), C 66.64, H 7.87, N 5.76%.

### 4.3. Receptor Binding Assays

*Drugs and reagents.* Radioligands [^3^H]-DAMGO, [^3^H]-DPDPE and [^3^H]-(+)-U69,593 were purchased by Perkin-Elmer Life Sciences (Boston, MA, USA). Naloxone hydrochloride was purchased from Tocris Bioscience (Bristol, UK). 

*Competitive Radioligand Binding Assay.* MOR, DOR and KOR binding experiments were performed on rat or guinea pig brain membranes according to the experimental protocol described by Spetea et al. [17], as reported elsewhere [18]. Protein concentration was determined by Lowry’s method using bovine serum albumin as standard [19]. Binding experiments at MOR and DOR were carried out by incubating 0.4 mg/mL and 0.5 mg/mL of rat brain membrane proteins, respectively for 45 min at 35 °C either with 1 nM [^3^H]-DAMGO (48.4 Ci/mM) or 2 nM [^3^H]-DPDPE (52.7 Ci/mM) in 50 mM Tris-HCl (pH 7.4). Regarding KOR binding assays, guinea pig brain membranes (0.5 mg/mL) were incubated for 30 min at 30 °C with 1 nM [^3^H]-(+)-U69,593 (42.69 Ci/mM). Test compounds were added in concentration ranging from 10^−5^ to 10^−11^ M. Nonspecific binding was assessed in the presence of 10 μM of unlabeled naloxone. The reaction was terminated by filtering the solution through Whatman GF/B glass fiber filters, which were presoaked for 1 h in a 0.5% polyethylenimine solution. Filters were washed with ice-cold buffer (2 × 4 mL), dried, soaked in 4 mL of “Ultima Gold MV” scintillation cocktail and counted on a Beckman LS 6500 liquid scintillation counter. 

*Data analysis.* K_i_ values were calculated using the EBDA/LIGAND program (version 4) purchased from Elsevier/Biosoft (Cambridge, UK).

### 4.4. cAMP Accumulation Assay

*Cell culture.* HEK293 cells stably expressing either the EE-tagged MOR or the flag-tagged KOR [20,21,22] were grown in Dulbecco’s modified Eagle’s medium (DMEM) supplemented with 10% fetal calf serum, 2 mM glutamine, 100 U/mL penicillin, and 0.1 mg/mL streptomycin under 5% CO_2_ at 37 °C. 

*Measurements of cAMP accumulation.* Measurements of AC activity were performed as described by Papakonstantinou et al. [22] or the GloSensor cAMP Assay (Promega) following the Manufacturer’s instructions. The generation of cAMP was assessed in response to treatment of the cells with various concentrations of the appropriate ligand (10 pM–50 μΜ) using 50 μΜ forskolin at 37 °C. Values are means ± SD of triplicate determinations from three independent experiments. Analysis of the data was performed using Origin 7.5 software (OriginLab Corporation, Northampton, MA, USA).

### 4.5. In Vivo Pharmacology

*Animals.* Male Swiss CB1 mice (Envigo Laboratories, S. Pietro al Natisone (UD), Italy) weighing 25–30 g were housed six to a cage. Animals were kept at a constant room temperature (25 ± 1 °C) under a 12:12 h light and dark cycle with free access to food and water. Each mouse was used for only one experiment. Experimental procedures were approved by the Local Ethical Committee (IACUC) and conducted in accordance with international guidelines as well as European Communities Council Directive and National Regulations (CEE Council 86/609 and DL 116/92).

*Nociceptive Test.* Nociception was evaluated by the radiant heat tail-flick test [11,23]. Briefly, it consisted of irradiation of the lower third of the tail with an infrared source (Ugo Basile, Comerio, Italy). The day before the experiment, mice were habituated to the procedure for measuring nociception threshold. Experiments were performed at room temperature (25 ± 1 °C). The basal pre-drug latency was established between 3 and 5 s and was calculated as the average of the first three measurements, which were performed at 5 min intervals. A cut-off latency of 15 s was established to minimize damage to the tail. All tested compounds were dissolved in pyrogen-free isotonic saline (Baxter Healthcare, Deerfield, IL, USA) and DMSO (5%) and were administered to mice ip. Post-treatment tail flick latencies (TFLs) were determined at 30, 45, 60, 75 and 90 min after ip injection. 

*Statistical Analysis.* All values were presented as means ± SD. Intergroup comparisons were assessed using an initial two-way analysis of variance (ANOVA) followed by Duncan’s multiple range post-hoc test. Differences were considered significant when * *p* < 0.05. Effective dose-50 (ED_50_) values were calculated using least-squares linear regression analysis followed by calculation of 95% confidence limits (95% CL) by the method of Bliss [12,24].

## 5. Conclusions

This study expands the understanding on the importance of *N*-substituent structural variations in opioid receptor profile of *cis*-(−)-*N*-normetazocine derivatives and identify compound **5c** as a new MOR agonist useful for the development of novel opioid analgesics for pain treatment.

## Figures and Tables

**Table 1:** Opioid receptor binding affinity of compounds **5a**–**d** and **6a**–**d**.

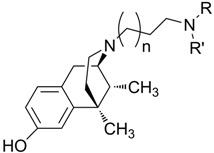						
Compound	n	R	R′	K_i_ (nM) ± SEM ^a,b^		
				MOR	DOR	KOR
**5a**	0	H	Ph	83 ± 30	270 ± 10	100 ± 5.0
**5b**	0	H	C_6_H_11_	8.3 ± 0.8	70 ± 2.0	19.8 ±0.9
**5c**	0	CH_3_	Ph	6.1 ± 0.5	147 ± 5.7	31 ± 1.3
**5d**	0	C_2_H_5_	Ph	160 ± 7.0	411 ±14	28.7 ± 1.0
**6a**	1	H	Ph	7.0 ± 0.6	117 ± 5.2	71 ± 2.0
**6b**	1	H	C_6_H_11_	48 ± 1.4	>5000	126 ± 4.5
**6c**	1	CH_3_	Ph	68 ±1.6	>5000	94 ± 3.5
**6d**	1	C_2_H_5_	Ph	86 ± 3.0	1060 ± 152	56 ±1.7
**LP1**				0.83 ± 0.05	29.1 ± 1.0	110 ± 6.0
**DAMGO**				1.16 ± 0.1	-	-
**U50,488**				-	-	0.34 ± 0.1
**Naltrindole**				-	1.13 ± 0.1	

^a^ Values are means ± SEM of three separate experiments, each carried out in duplicate. ^b^ K_i_ values were obtained as [^3^H]DAMGO displacement for MOR, [^3^H]DPDPE displacement for DOR, and [^3^H]U69,593 displacement for KOR.

**Table 2:** Effect of compounds **5a**–**d** and **6a**–**d** on cAMP accumulation by the KOR and MOR.

Compound	IC_50_ (nM) ± SD ^a,b^		I_max_ (%) ± SD ^c^	
	MOR	KOR	MOR	KOR
**5a**	55.3 ± 7.0	1000 ± 65	60 ± 4	-
**5b**	74.0 ± 3.5	180 ± 50	28 ± 1	58 ± 4
**5c**	11.5 ± 2.5	ND ^d^	72 ± 5	-
**5d**	66 ± 1.3	ND	55 ± 3	-
**6a**	7.4 ± 1.1	1400 ± 69	50 ± 3	53 ± 4
**6b**	ND	>5000	-	44 ± 3
**6c**	21.61 ± 3.5	ND	50 ± 3	-
**6d**	9.51 ± 2.0	ND	40 ± 2	-
**LP1**	4.8 ± 0.5	-	73 ± 3.8	-
**DAMGO**	3.18 ± 0.3	-	73 ± 0.3	-
**U50,488**		0.82 ± 0.03		68 ± 5

^a^ Agonist properties of compounds in the inhibition of forskolin-stimulated cAMP accumulation by MOR and KOR. The inhibition of cAMP accumulation was measured as described in Section 4.3. ^b^ IC_50_ value is the concentration of the compound needed to produce half maximal inhibition, with all values presented as the average ± SD of triplicate determinations from three independent experiments. ^c^ IC_max_ value is the maximal percent inhibition obtained with the compound. ^d^ ND, not determined.
